# Male Responses to Conspecific Advertisement Signals in the Field Cricket *Gryllus rubens* (Orthoptera: Gryllidae)

**DOI:** 10.1371/journal.pone.0016063

**Published:** 2011-01-20

**Authors:** Yikweon Jang

**Affiliations:** Department of Life Sciences and Division of EcoScience, Ewha University, Seoul, Republic of Korea; University of Maribor, Slovenia

## Abstract

In many species males aggregate and produce long-range advertisement signals to attract conspecific females. The majority of the receivers of these signals are probably other males most of the time, and male responses to competitors' signals can structure the spatial and temporal organization of the breeding aggregation and affect male mating tactics. I quantified male responses to a conspecific advertisement stimulus repeatedly over three age classes in *Gryllus rubens* (Orthoptera: Gryllidae) in order to estimate the type and frequency of male responses to the broadcast stimulus and to determine the factors affecting them. Factors tested included body size, wing dimorphism, age, and intensity of the broadcast stimulus. Overall, males employed acoustic response more often than positive phonotactic response. As males aged, the frequency of positive phonotactic response decreased but that of the acoustic response increased. That is, males may use positive phonotaxis in the early stages of their adult lives, possibly to find suitable calling sites or parasitize calling males, and then later in life switch to acoustic responses in response to conspecific advertisement signals. Males with smaller body size more frequently exhibited acoustic responses. This study suggests that individual variation, more than any factors measured, is critical for age-dependent male responses to conspecific advertisement signals.

## Introduction

Males of many acoustic species such as insects and anurans may form breeding aggregations and produce advertisement signals to attract females. Neighboring males in an aggregation are often in intense competition to attract females. The acoustic signal from a male is critical for attracting females as well as for competing with neighboring males [1], [2], [3]. Although the acoustic signals may have these dual functions, recipients of the acoustic signals are much more likely neighboring males most of the time, rather than conspecific females, especially in a population with a male-biased operational sex ratio. When neighboring males in close proximity interact with each other acoustically, chorus structure [4], [5], [6] and spatial distance between adjacent signalers [7], [8], [9] are mediated by the response to neighbors' signals.

In addition to the structure of breeding aggregations, male responses to conspecific advertisement signals also affect male mating tactics. In singing insects and anurans, two alternative mating tactics have been reported: calling and becoming a satellite [10], [11], [12], [13]. In the calling tactic, males maintain a calling territory and produce calling songs regularly to attract conspecific females. In the satellite tactic, males remain silent and stay close to singing males to intercept females orienting to the singing males. There is evidence that the choice between alternative mating tactics typically employed by a singing insect or anuran may depend on characteristics of the conspecific advertisement signals [14]. Thus, male response to conspecific advertisement signals can structure the spatial and temporal organization of the breeding aggregation and determine male mating tactics.

In response to conspecific advertisement signals, males of acoustic insects and anurans may employ two types of response: (1) phonotactic response in which males move toward the source of the signal and (2) acoustic response in which males respond to the signal by producing their own acoustic signals. Currently, it is unclear what factors and circumstances may determine the type and frequency of male response. In acoustic insects and anurans, male responses to conspecific advertisement signals may be influenced by both intrinsic and extrinsic factors. Intrinsic factors include age, morphology, or nutritional status. Male call characteristics change during the course of adult lives [15], [16], [17], [18], and theoretical studies have suggested age-specific female preferences during mate choice [19], [20], [21], [22]. In acoustic insects where mate choice is mediated by long-distance calling, there is evidence of female preference for younger males in some cases [23] and older males in others [18], [24], [25]. In either case, males may selectively alter the type and frequency of their responses to conspecific advertisement signals as they age. When females prefer males of a certain age group, males of this group may exhibit acoustic response to neighboring males, rather than positive phonotactic response. Males relying on acoustic response may simultaneously attract responsive females and repel competitive neighboring males. Positive phonotaxis in response to conspecific advertisement signals may be also age dependent. For example, newly eclosed males that have no calling territories yet may have to join a breeding aggregation.

Morphology can have direct effects on male response to conspecific advertisement signals in relation to both mating tactics and the nature of response. In crickets, males with smaller body size often produce signals with low intensity [26], [27]. If smaller males produce sounds in response to conspecific advertisement signals, they may risk being displaced by larger males. There is evidence that smaller males typically use less confrontational mating tactics, such as becoming satellites, in their response to conspecific signalers [10], [11], [28]. Three hypotheses may explain male positive phonotaxis in response to conspecific advertisement signals: satellite male, aggressive displacement, and chorus attendance. The satellite male hypothesis states that males, especially those with smaller body size, may employ phonotaxis in response to conspecific advertisement signals to became satellites [29]. A corollary of this hypothesis is that males with smaller body size may rely more on positive phonotaxis rather than on acoustic response. The aggressive displacement hypothesis predicts that males with larger body size may rely on positive phonotaxis to displace competitors for suitable calling sites [29]. Such males would enjoy the advantage in resource holding potential in aggressive interactions [30], [31], [32]. Alternatively, the chorus attendance hypothesis states that males join a calling aggregation to produce advertisement signals. In this hypothesis, body size may not be correlated with positive phonotaxis.

In some cricket species, adults exhibit wing dimorphism: macroptery and microptery [33], [34], [35]. Macropterous individuals have hindwings that are longer than their forewings and can fly for dispersal, whereas micropterous individuals have hindwings shorter than their forewings and are flightless. In *G. rubens*, macropterous females typically begin ovipositing earlier and oviposit more eggs than micropterous females [36]. In *G. firmus*, micropterous males are more likely to spend time calling and attract females than macropterous males [37], [38], [39]. Due to the differences in dispersal ability and time spent calling, micropterous and macropterous individuals of *G. rubens* may differ in the type and frequency of male response to conspecific advertisement signals.

In addition to intrinsic factors, extrinsic factors, such as the signal intensity of neighbors, the density of competitors, predation risk, and female availability, may determine the type and frequency of male response to conspecific advertisement signals. In acoustic animals, call intensity may be used as a proxy for distance to the signaler and thus used as a cue for mediating spacing among signaling males [8], [26], [40], [41]. If the signal is strong, then the calling neighbor is close and is a direct competitor for female attraction. In response to increased call intensity, males may employ acoustic response by making temporal [14], [42], [43], spectral [14], [44], [45], [46], [47], or amplitude [45] adjustments to the advertisement signals or by producing qualitatively different aggressive signals [48], [49]. Positive phonotaxis is also an option. Males may orient toward the signal to displace the signaler if the signal intensity is low or to become satellites if the signal intensity is high. There is a possibility that the type of male response is not independent of signal intensity. For example, males may respond to a low intensity signal with acoustic response and respond to a high intensity signal with positive phonotaxis, or vice versa.

*Gryllus rubens* Scudder (Orthoptera: Gryllidae) is one of the most abundant field cricket species in the southeastern United States. Males often aggregate and produce calling songs to attract females. Adults exhibit wing dimorphism: both micropterous and macropterous individuals occur together in a population. Here I used a synthetic acoustic stimulus with the characteristics of *G. rubens* advertisement songs to investigate male response over three age classes. The experimental period covering the three age classes may represent most of the singing activity of adult crickets [15], [50]. I measured both phonotactic and acoustic responses against the conspecific advertisement stimulus. The goal of this study was to understand the type and frequency of male response to the conspecific advertisement stimulus and to determine the factors affecting male responses. Along with documenting age-specific male responses, I tested *post hoc* hypotheses about male positive phonotaxis to the conspecific advertisement signals by examining the relationship between body size and the frequency of positive phonotaxis.

## Methods

### Ethical treatment of animals

Ethical approval was not required for the field cricket *Gryllus rubens*, the subject in this study, because *G. rubens* is not listed as an endangered species. In fact, this species is one of the most abundant field cricket species in eastern United States and is found numerously almost everywhere in their natural range. However, maintenance of field cricket stocks in the laboratory and playback experiments with the study subject were carried out in strict accordance with the recommendations of Animal Care Quality Assurance in the University of Missouri-Columbia.

### Study species and population

*G. rubens* has a bivoltine life cycle, with adults occurring in spring and fall [35]. They overwinter as juveniles and are typically found in lawns, pastures, and roadsides. In south Florida, however, adults and middle-sized to large juveniles can be found throughout the year. Phylogenetic analyses based on DNA sequences suggest that *Gryllus* species in eastern North America may be divided into four clades characterized by different life history traits, and that *G. rubens* forms a separate clade with *G. fultoni* and *G. pennsylvanicus* [51], [52], [53]. *G. rubens* is morphologically distinguished from other *Gryllus* species in the southeastern United States by the color pattern of its forewings [54].

Adults of *G. rubens* (31 males and 34 females) were caught in Goreville, Illinois, USA, in April 2004. Field-caught females were placed on wet sand to collect their eggs. The progeny of these crickets were reared from the first instars to adults in plastic bins (33×50×29 cm) with holes on the side for ventilation as well as adequate shelter inside. Each plastic bin housed fewer than 200 individuals. Both juvenile and adult crickets were provided *ad libitum* with cricket chow and lettuce. Newly emerged adults were removed within 24 h of the final molt from the stock population and were housed in individual containers (12×12×9 cm) to ensure that all crickets used for this study were virgin [55]. All experiments were conducted on the first-generation offspring of the field-caught females. All crickets were maintained at 23±1°C with a 14:10 h, light:dark photoperiod during development. The age of crickets was measured as the number of days from the final molt to the experiment.

### Male calling songs

To estimate the parameters of calling song characters for use as the test stimulus, I recorded calling songs of 25 males that were caught in the field. I failed to record calling songs of the remaining six. Field-caught adult males were held at least seven days from capture before recording their calling songs. All recordings and playback trials were conducted in a temperature-controlled anechoic chamber (3×3×2 m) at the University of Missouri-Columbia. To record calling songs, a male cricket housed in an individual container with a screened lid was allowed to acclimate to the anechoic chamber for at least 30 min. A Sennheiser microphone (ME 66 shotgun head + K6 powering module; frequency response: 50–20000 Hz ±2.5 dB) was placed 58 cm directly above the container. Output from the microphone was fed into a USB audio interface (Edirol Corp. Model UA-5, 20 to 20,000 Hz: +0dB/−2dB), operating at a sampling rate of 44.1 kHz. The signal from the audio interface was output into a PC. Each singing cricket was recorded for 2 min. The temperature of the room was measured using a thermocouple probe (Model 450-AKT, Omega Engineering, Inc., Stamford, CT) that was placed in the chamber near a calling male. The temperature of the anechoic chamber was maintained at 23±1°C during recording.

Cricket recordings were analyzed using Raven 1.1 (Cornell Laboratory of Ornithology, Ithaca, NY). Males of *G. rubens* produce a trilled calling song in which a continuous train of sound pulses is repeated [56], [57]. At 25°C, *G. rubens* trills at approximately 56 pulses/s [58], [59], [60], [61]. Pulse duration (PD) is defined as the time between the start and the end of a pulse. Pulse period is the time from the start of one pulse to the start of the subsequent pulse, and pulse rate (PR) is the inverse of the pulse period. Carrier frequency (CF) refers to the frequency with the most acoustic energy. Trill duration (TD) is the time between the start and the end of a trill. Trill rate (TR) is the inverse of the trill period that is the time from the start of one trill to the start of the subsequent trill. At least fifty consecutive pulses were examined for PR, PD, and CF in a trill. Estimation of TD and TR were based on the entire 2-min recordings for statistical analyses.

### Playback experiment

Based on the mean values of calling song characters recorded in the laboratory (Table 1), the test stimulus was synthetically generated using custom-designed software with 16-bit resolution and 44.1 kHz sampling rate [62], [63]. The pulse shape was constant for all stimuli with a 10% linear rise time, 80% flat time, and 10% fall time. Each trill consisted of 211 pulses. The test stimulus was 6 min and 34 s long and was written on a compact disc for playback.

**Table 1:** Characteristics of *G. rubens* calling songs (*n* = 25) on which the parameters of the test stimulus were based.

Characters	Mean	SD	Min	Max
Pulse rate (s^−1^)	40.424	3.693	33.225	49.381
Pulse duration (ms)	10.4	2.0	5.5	14.6
Trill rate (s^−1^)	0.217	0.095	0.040	0.436
Trill duration (s)	5.210	5.075	1.636	26.496
Carrier frequency (Hz)	4411	248	4053	4850

Calling songs of field-caught *G. rubens* males were recorded in the laboratory at 23±1°C. See the main text for recording and song analysis procedures.

All playback experiments were conducted in an octagonal arena that measured 50 cm from the center to the corner [63]. The arena was built on a plywood base covered with a thin layer of carpeting. The boundary of the arena was a 12 cm-high acoustically-transparent screen. The test stimulus was played back using a Sony CD player (Model No. D-SJ301, Japan), and the output from the CD player was sent to an attenuator-amplifier (TDL Technology, Inc. Model 439; Las Cruces, New Mexico, USA; less than −0.1 dB at 10 Hz at all gains and −0.5 dB at 124 kHz at +20 dB). The signal from the attenuator-amplifier was sent to one of two tweeters (Dayton #275-100 Euro series textile tweeter; Springboro, OH, USA; flat frequency 2,000-20,000 Hz), placed at floor level outside the arena wall (55 cm from the center of the arena). The angular separation between the two tweeters was 120°. Choice of a tweeter for playback trials was switched daily to control for any phonotactic bias that might be present in the arena. During the trials, the anechoic chamber was lighted with three red 25-W incandescent bulbs.

Male crickets to be tested were drawn haphazardly from the stock population after eclosion. Only intact males were used for the playback experiment. Each trial began with a male cricket placed under an opaque plastic cover (250 ml) located in the center of the arena. The plastic cover had holes for sound permeation. The cricket was placed in the arena for 1 min without any sound to acclimate and then was exposed to the test stimulus broadcast from one of the tweeters for 1 min. Then the cricket was released by removing the cup, and 5 min was allowed for a response to the stimulus. I scored (1) an acoustic response if a male produced a song during the trial and (2) a positive phonotactic response if a male oriented towards the tweeter that broadcast the test stimulus. Trials were terminated after 5 min or when test males displayed positive phonotaxis. A positive phonotactic response was recorded if a male oriented toward the tweeter and touched the wall of the arena directly in front of the loudspeaker broadcasting the test stimulus or if a male made a “double reversal” movement in front of the tweeter [63]. That is, a cricket sometimes ran toward the arena wall after release without orientation and then walked along the wall. A male cricket was described as displaying a “double reversal” if it reversed its walking direction in front of the tweeter broadcasting the test stimulus twice in a row while in close proximity to the tweeter; each reversal of direction had to cross the line between the tweeter and the male release point. The double reversal movement is a local search and occurs when an individual is very close to the object for which it is searching [64]. A score of “no response” was recorded if the male left the release point but did not orient toward the test stimulus or did not make a double reversal movement, or if the male failed to leave the release point within the 5-min trial.

Acoustic responses involve the production of sound by forewing stridulation. To record acoustic responses during trials, a Sennheiser microphone (ME 66 shotgun head + K6 powering module, frequency response: 50–20000 Hz ±2.5 dB) was placed 40 cm directly above the center of the arena. Output from the microphone was fed into a Sony PCM-M1 DAT recorder operating at a sampling rate of 44.1 kHz. Cricket signals were analyzed using Raven 1.1 (Cornell Laboratory of Ornithology, Ithaca, NY) installed on a PC. I measured PR, PD, CF, TR, and TD from recordings. Measurements of PR, PD, and CF were only conducted during the intervals between trills of the broadcast stimulus where no sound was present.

Each cricket was tested four times over two consecutive days at a given age using the test stimulus with each of four intensity levels: 63, 69, 75, and 81 dB SPL (0 dB  = 20 µPa). Two trials separated by at least two hours were conducted each day. The order in which the respective intensity levels were tested was determined randomly for each age. The intensity level of the broadcast stimulus was calibrated at the center of the arena using a Brüel & Kjær (Model 2209; Nærum, Denmark) sound level meter at the peak setting. To understand how male responses changed with age, the playback experiment consisting of four intensity trials was conducted on days 8–9, 15–16, and 22–23 for each cricket. After completing the playback experiments, male crickets were measured once for head width and hind femur length using a digital Vernier caliper to the nearest 0.05 mm. Head width was defined as the distance between the outer edges of the compound eyes. A right hind leg was removed from the thorax and placed on its side. Hind femur length was measured from the base of the femur to the joint with the tibia.

### Statistical analyses

A total of 24 micropterous and 25 macropterous *G. rubens* males was used for this study. Two males died before the age 22 trials, each yielding 8 responses. One male died before the final trial, yielding 11 responses. In all, there were 579 trials. The average number of trials per individual was 11.82±0.808 (mean ± S.D.).

Because male crickets were repeatedly subject to phonotactic trials, and because age might affect male responses [65], [66], [67], I used a generalized linear mixed model (PROC GLIMMIX) in SAS (ver. 9.1; The SAS Institute; Cary, North Carolina, USA) to understand factors that affected the type and frequency of male response. Individual identity was a random factor with a binomial error term. I used the forward selection procedure to find the best combination of variables to fit for both positive phonotactic and acoustic responses. Predictor variables were intensity of the stimulus, age, wing morph, and body size. To estimate body size, I conducted principal components analysis for head width and hind femur length. Only the first component of this analysis (eigenvalue  = 1.605) had an eigenvalue greater than one, and this component explained 80.3% of the variation. Accordingly, I used the first component as a measure of body size. The response variables were positive phonotactic and acoustic responses. Male phonotactic responses were categorized as 1 for positive responses or 0 for all other responses. Acoustic responses were scored as 1 if a male produced a song or 0 if he remained silent.

Because positive phonotactic and acoustic responses were measured up to 12 times, I tried to measure whether individual crickets were consistent in their choice of response over three age classes and across four intensity levels. In the analyses of the generalized linear mixed model, the covariance for individual identity (among-individual variance) and the residual (within-individual variance) were estimated for each type of response. The repeatability of either type of response was calculated as the ratio of the covariance estimate for individual identity to the sum of covariance estimate for individual identity and the residual estimate [68], [69]. To understand the changes in male acoustic signals in response to the broadcast stimulus, I developed a multivariate general linear model (GLM) for five acoustic characters. The predictor variables of this analysis were individual, age and intensity.

## Results

### Morphological characteristics

Head width of the macropterous individuals (*n* = 25) was 5.117±0.2514 mm (mean ± SD), and that of the micropterous individuals (*n* = 24) was 5.010±0.3235 mm. Hind femur length of the macropterous individuals was 10.614±0.4559 mm, and that of the micropterous individuals was 9.982±0.5900 mm. Hind femur length was significantly different (two-tailed *t*-test, *t* = −4.179,*df*  = 47, *P*<0. 001) between macropterous and micropterous individuals, whereas head width was not (two-tailed *t*-test, *t* = −1.307, *df*  = 47, *P* = 0.200).

### Male responses

**Figure 1:**
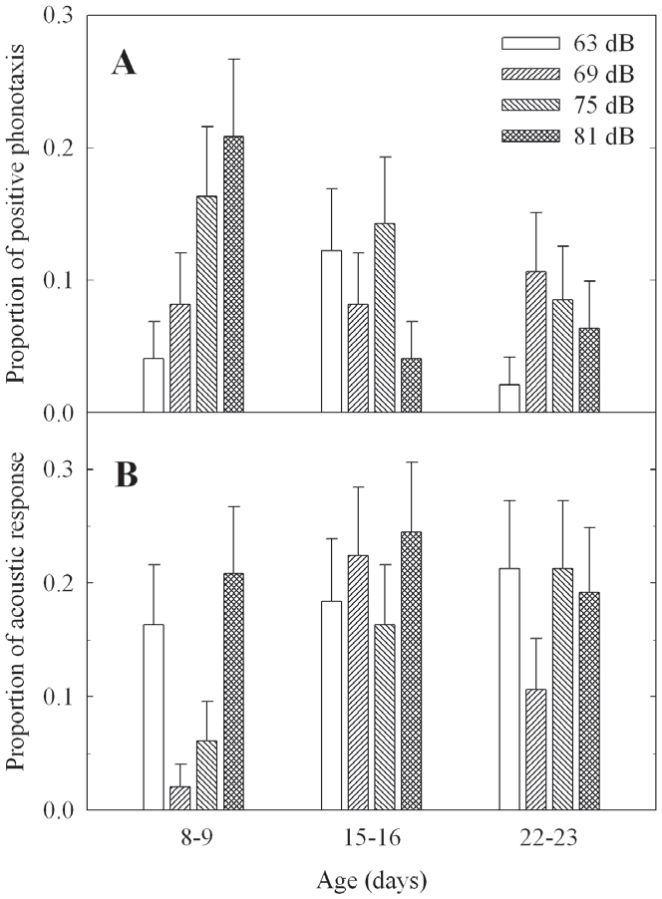
Proportions of positive phonotaxis (A) and acoustic response (B) toward the test stimulus in the playback experiment. At a given age, males were tested with four intensity levels (0 dB SPL  = 20* µ*Pa) over a two-day trial period (*n* = 49 males and 579 trials). See text for experimental methods.

Of the 49 *G. rubens* males, only 15 exhibited both positive phonotactic and acoustic responses to playbacks of the broadcast stimulus at least once over three age classes, and eight males never showed either response. Overall, acoustic response occurred more often than phonotactic response (general loglinear analysis, χ^2^ = 4.790, *df*  = 1, *p* = 0.029; Table 2). In 12 trials (four intensity levels and three age groups), 50% and 37% males did not show positive phonotactic and acoustic responses, respectively. Furthermore, at any given age and stimulus intensity level, no more than 25% of individuals responded acoustically or phonotactically (Fig. 1).

**Table 2:** Contingency table of positive phonotactic and acoustic responses across three age classes and four intensity levels in the playback experiments.

	Nophonotaxis	Positive phonotaxis	Total
No acoustic response	431	52	483
Acoustic response	92	4	96
Total	523	56	579

Results of the macropterous and micropterous individuals were combined (*n* = 49 males). See text for description of behaviors.

The analysis of the generalized linear mixed model with forward selection determined that age was a significant factor affecting positive phonotactic responses and that age and body size both significantly affected acoustic responses (Table 3). Although age was a significant factor for both types of responses, the direction of the age effect differed between them. The proportion of positive phonotactic responses peaked at age 8–9 d and decreased with age, but the proportions of acoustic responses were higher at ages 15–16 d and 22–23 d than at age 8–9 d (Table 3). The ratio of acoustic to positive phonotactic responses was 0.92 at 8–9 d of age, but increased to 2.11 at 15–16 d and to 2.62 at 22–23 d. Intensity seemed to be an important factor affecting positive phonotaxis. The proportions of positive phonotactic responses were higher at intensity levels 75 dB and 81 dB than at intensity levels 63 dB and 69 dB. There was an interaction between age and intensity levels of the stimulus for positive phonotactic responses (Table 3). Proportions of positive phonotaxis were higher when males were younger and the stimulus intensities were higher (Fig. 1). The frequency of acoustic responses decreased as body size increased (i.e., smaller males sang more), but there was no apparent relationship between positive phonotactic responses and body size. Wing dimorphism was not a significant factor affecting either type of male response. Male crickets consistently responded to the test stimulus with positive phonotaxis or song production across three ages and four intensity levels. The repeatability estimate of the positive phonotactic response was 0.7610, and that of the acoustic response was 0.6774.

**Table 3:** The best models for positive phonotactic (a; -2 Res Log Pseudo-likelihood  = 3143.73, Generalized chi-square  = 341.47, Generalized chi-square/*df*  = 0.59) and acoustic (b; -2 Res Log Pseudo-likelihood  = 2916.42, Generalized chi-square  = 422.65, Generalized chi-square/*df*  = 0.73) responses determined by a generalized linear mixed model with forward selection.

	Estimate	Standard error	*df*	*F*	*P*
(a) Positive phonotactic response					
Intercept	−2.7722	0.2479	48		
Age	−0.2540	0.1229	527	−2.07	0.0393
Intensity	0.2128	0.1219	527	1.74	0.0816
Age × Intensity	−0.2650	0.1223	527	−2.17	0.0306
(b) Acoustic response					
Intercept	−1.9886	0.2169	47		
Age	0.2567	0.1058	529	2.43	0.0155
Body size	−0.4413	0.2192	529	−2.01	0.0445

### Variation in characteristics of acoustic responses

**Figure 2:**
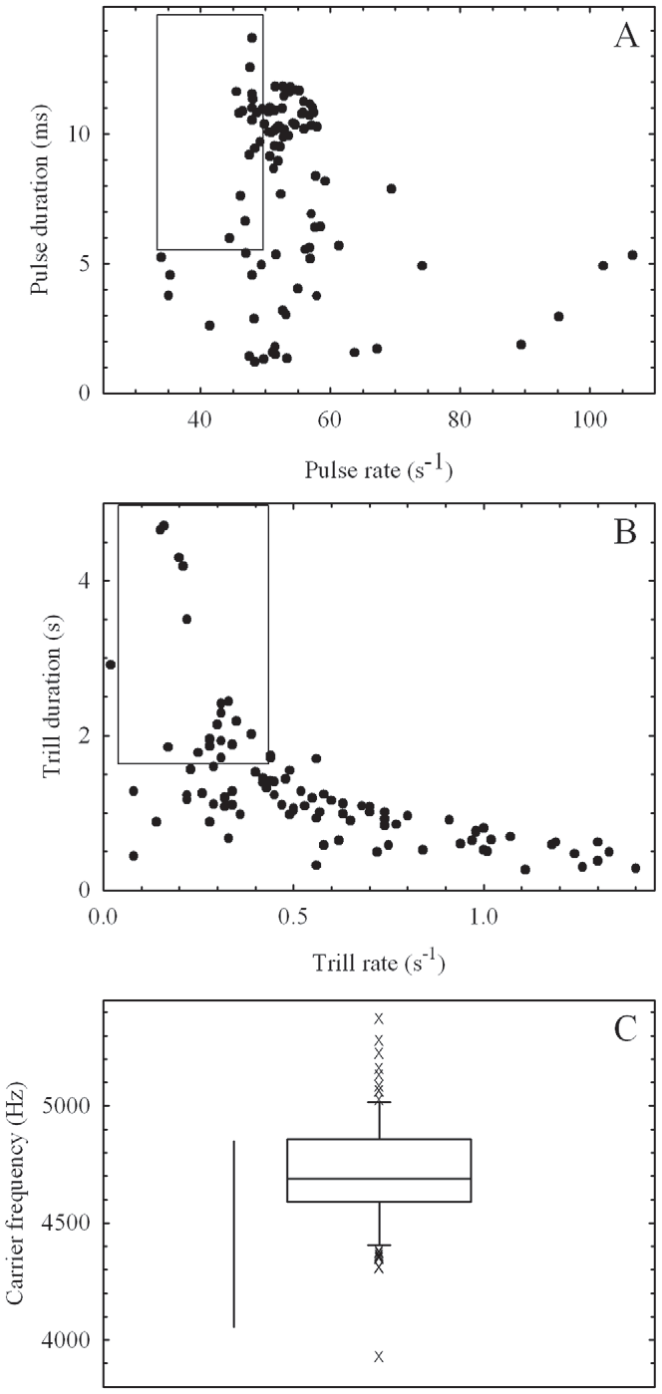
Distributions of song characters produced in the playback experiment (*n* = 96). In the top panel (A), pulse rate is plotted against pulse duration, and in the middle panel (B), trill rate is plotted against trill duration. Rectangular areas in both panels represent the ranges of respective characters in calling songs of field-caught males (see Table 1). The box plot in the bottom panel (C) shows the distribution of carrier frequency of songs produced in the playback experiment. Top, middle, and bottom lines of the boxes indicate quartiles, and the upper and lower whiskers indicate 90th and 10th percentiles, respectively. X denotes an outlier. The vertical bar represents the range of carrier frequency in calling songs of field-caught populations (see Table 1).

In 96 out of 579 playback trials, males produced sounds in response to the broadcast stimulus. Compared to the characteristics of calling songs (Table 1), pulses and trills of songs produced during playback trials were generally shorter in duration and were delivered at higher rates, beyond the natural ranges of calling songs (Fig 2A and 2B). The mean carrier frequency (4716±233 Hz; mean ± S.D.) was significantly different from that of the field population (see Table 1; independent sample t-test; *t* = 5.760, *df*  = 119, *P*<0.001; Levene's test for equality of variances, *F* = 0.451, *P* = 0.503), but the distribution of carrier frequency produced during playback trials was generally overlapping with that of the calling songs (Fig. 2C). Results of the multivariate GLM revealed that individual (Wilks' Lambda  = 0.001, *F* = 5.668, *P*<0.001) was the only significant factor for variation in characteristics of acoustic responses (Table 4). Age (Wilks' Lambda  = 0.770, *F* = 1.477, *P* = 0.158) and intensity of the broadcast stimulus (Wilks' Lambda  = 0.867, *F* = 0.518, *P* = 0.928)were not significant factors.

**Table 4:** Result of the analysis of variance to test for variation in properties of acoustic responses over three age classes and four intensity levels.

Source	Character	df	MS	F	P
Individual	Pulse rate	33	213.79	3.253	<0.001
	Pulse duration	33	28.86	10.269	<0.001
	Trill rate	33	0.21	3.742	<0.001
	Trill duration	33	1.86	8.462	<0.001
	Carrier frequency	33	111019.81	4.957	<0.001
Age	Pulse rate	2	173.68	2.642	0.080
	Pulse duration	2	5.73	2.040	0.139
	Trill rate	2	0.04	0.714	0.494
	Trill duration	2	0.48	2.195	0.121
	Carrier frequency	2	45074.13	2.013	0.143
Intensity	Pulse rate	3	38.27	0.582	0.629
	Pulse duration	3	3.09	1.101	0.356
	Trill rate	3	0.04	0.756	0.523
	Trill duration	3	0.217	0.986	0.406
	Carrier frequency	3	14456.45	0.645	0.589
Error	Pulse rate	57	65.73		
	Pulse duration	57	2.81		
	Trill rate	57	0.05		
	Trill duration	57	0.22		
	Carrier frequency	57	22396.13		

Response variables were pulse rate, pulse duration, trill rate, trill duration, and carrier frequency (*n* = 96).

## Discussion

The results of the playback experiment revealed age-dependent male responses to the conspecific advertisement stimulus in the field cricket *G. rubens*. As males aged, the primary response to the conspecific advertisement signals switched from positive phonotaxis to song production. The increase in use of the acoustic response with age seems to coincide with a period of high singing activity and increased sexual attractiveness in crickets. In the Texas field cricket, *G. texensis*, middle-aged males, especially those aged between 10 and 17 d, called significantly more often than young and very old males [15]. By employing the acoustic response, males may attract conspecific females for mating and maintain spacing against neighboring rivals singing at the same time. Thus, the results of the playback experiments were largely consistent with the prediction that males of a preferred age may rely on acoustic response.

Female crickets typically rely on phonotaxis for mate location and mate choice, but the function of male phonotaxis is usually difficult to determine [29]. Body size was not a significant factor for positive phonotaixs in this study, a result that supports neither the satellite male hypothesis nor the aggressive displacement hypothesis. Rather, this result is consistent with the chorus attendance hypothesis, in which males, irrespective of size, may orient toward the conspecific advertisement signals in order to join a calling aggregation. The fact that the frequency of positive phonotactic responses peaked at age 8–9 d and decreased with age may also support the chorus attendance hypothesis. Male crickets at the early stage of their adult life may use phonotaxis to locate suitable sites for reproduction. For example, in the wood frog *Rana sylvatica*, which has a short breeding period, males may use phonotaxis to locate short-lived breeding aggregations [70]. The presence of other male crickets advertised by production of calling songs may indicate the locations of such suitable sites to which males orient. However, both satellite male and aggressive displacement hypotheses cannot be ruled out in this study, because body size alone may not be a good predictor for male phonotaxis.

In the satellite male hypothesis, a smaller male may rely on phonotaxis to parasitize a calling male [14], [71], whereas, a male employing the calling tactic may use phonotaxis to locate a calling individual, leading to aggressive behavior in which the male challenges the signaler in a physical contest [48], [72], [73]. In a choice of stimuli with different degrees of attractiveness to females, smaller males chose the female-preferred signals more frequently than did larger males in the house cricket *Acheta domesticus* [74], the spadefoot toad *Spea multiplica* [75], and the tree frog *Hyla cinerea* [71]. In this study, the frequency of positive phonotaxis at age 8–9 d was particularly high toward the stimulus with higher intensities, features of which might be attractive to females. This provides limited support for the satellite male hypothesis to explain male phonotaxis to a conspecific advertisement signal in *G. rubens*. There is also evidence for the aggressive displacement hypothesis. Leonard and Hedrick (2009) studied the relationship between fighting ability and male phonotaxis in *G. integer*. In that study, less attractive males did not preferentially approach female-preferred calls, but males that were likely to win contests preferentially approached female-preferred calls. In a separate field study of *G. campestris* in which a calling song was broadcast to burrows occupied by calling and non-calling males throughout the mating season, calling males at burrows performed phonotaxis more frequently compared with non-calling males at burrows [76], providing strong support for the aggressive displacement hypothesis. To understand the functions of male phonotaxis to conspecific advertisement signals, a number of factors such as fighting ability, female preference for calling songs, and male spacing would have to be characterized among a group of males with the natural range of variation in body size.

Song characters of the acoustic responses varied across individuals, but did not vary according to the intensity of the broadcast stimulus or male age. The characteristics of songs produced during playback trials were quite different from those of calling songs in *G. rubens*. Males responding to conspecific advertisement songs or stimuli typically increase the rate of the basic song structures such as pulses, notes, chirps, or calls in both acoustic insects [77], [78] and anuarans [42], [79], [80]. During the playback trials, males of *G. rubens* may be interrupted by the broadcast stimulus, resulting in higher rates of trills and shorter trill duration. Pulses delivered at higher rates and in shorter durations may be reminiscent of aggressive songs in crickets [81]. Male frogs typically alter the ratio of aggressive to advertisement calls in response to conspecific advertisement signals [8], [82]. Whether males respond to conspecific advertisement signals by introducing qualitatively different aggressive songs has not been investigated carefully in crickets.

In this study, smaller males were more likely to rely on acoustic response to the conspecific advertisement stimulus. If smaller males respond to conspecifics' advertisement signals with singing rather than with positive phonotaxis, they may enjoy asymmetry in residence when physically attacked by other males. The relationship between male body size and the tendency toward acoustic response to conspecific advertisement signals is largely unexplored. The results of a few studies testing this relationship indicate that body size seems to be unimportant as a predictor of male acoustic response [83], [84]. In those studies, however, the stimuli were presented to territorial males, a situation in which the value of residence may be more important than the asymmetry in resource holding potential [85], [86].

Individual male crickets were quite consistent in their response to the conspecific advertisement stimulus. Some individuals responded repeatedly by producing songs or orienting toward the conspecific stimulus over three age classes and across four different intensity levels. Furthermore, individual variation was the most important factor explaining the characteristics of songs produced during the playback trials. Individual variation in response to the broadcast stimulus was accentuated by the fact that some males never responded to the stimulus, and that at best a quarter of individuals responded at a specific age and at a given stimulus intensity level. Thus, there was a dichotomy in response to the conspecific advertisement signal, in which some individuals responded consistently, whereas the majority exhibited no response at all. A possible source of this dichotomy in response may be the experimental setting, in which males were exposed to the artificial stimulus without much time for establishing territory. However, the existence of males that do not exhibit any response at all to conspecific advertisement signals may not be unusual. In a recording study of male *G. rubens* aged between 10 and 17 d, some males never signalled, while others signalled for several hours each night [87].

Compared to female behaviors to conspecific advertisement signals, male responses have been studied far less often in acoustic communication of insects and anurans. Although this study demonstrated age-dependent male response to conspecific advertisment signals, several important questions remain. First, under what circumstances do males use positive phonotaxis in response to conspecific advertisement signals? Second, why do males consistently differ in their responses to conspecifics' signals? Third, for those males whose responses could not be clearly identified in this study, is their behavior in fact affected by conspecific advertisement signals? Finally, is age-dependent male response to the conspecific advertisement signals adaptive? If so, this should be described and incorporated into our understanding of the evolution of breeding organization and male mating tactics.
